# Genetically predicted iron status and life expectancy

**DOI:** 10.1016/j.clnu.2020.06.025

**Published:** 2021-04

**Authors:** Iyas Daghlas, Dipender Gill

**Affiliations:** a Harvard Medical School, Boston, MA, USA; b Department of Epidemiology and Biostatistics, School of Public Health, Imperial College London, London, UK

**Keywords:** Ferritin, Iron, Lifespan, Longevity, Mendelian randomization, Transferrin

## Abstract

**Background & aims:**

Systemic iron status affects multiple health outcomes, however its net effect on life expectancy is not known. We conducted a two-sample Mendelian randomization (MR) study to investigate the association of genetically proxied iron status with life expectancy.

**Methods:**

Using genetic data from 48,972 individuals, we identified three genetic variants as instrumental variables for systemic iron status. We obtained genetic associations of these variants with parental lifespan (*n* = 1,012,240) and individual survival to the 90th vs. 60th percentile age (11,262 cases and 25,483 controls). We used the inverse-variance weighted method to estimate the effect of a 1-standard deviation (SD) increase in genetically predicted serum iron on each of the life expectancy outcomes.

**Results:**

We found a detrimental effect of genetically proxied higher iron status on life expectancy. A 1-SD increase in genetically predicted serum iron corresponded to 0.70 (95% confidence interval [CI] −1.17, −0.24; *P* = 3.00 × 10^−3^) fewer years of parental lifespan and had odds ratio 0.81 (95% CI 0.70, 0.93; *P* = 4.44 × 10^−3^) for survival to the 90th vs. 60th percentile age. We did not find evidence to suggest that these results were biased by pleiotropic effects of the genetic variants.

**Conclusions:**

Higher systemic iron status may reduce life expectancy. The clinical implications of this finding warrant further investigation, particularly in the context of iron supplementation in individuals with normal iron status.

## 1 Introduction

Iron is vital to human health. Systemic iron status can be assessed using biomarkers such as serum iron, ferritin, transferrin (inversely related to iron status) and transferrin saturation. Levels of these biomarkers vary considerably between individuals, and can be altered through clinical intervention. Small changes in iron status have been suggested to have protective and detrimental effects on different disease processes [[1], [2], [3], [4]]. However, the net effect of varying systemic iron levels on life expectancy remains unclear. This is of clinical relevance, as up to 19% of the US population takes an iron supplement [5].

While observational studies have associated higher iron status with reduced life expectancy, such a study design is limited in its ability to draw causal inference due to the possibility of confounding and reverse causation [6]. For example, dietary patterns may influence both iron status and longevity, thereby inducing a spurious relationship between these two entities. Furthermore, biomarkers of iron status are influenced by acute and chronic inflammation, and may therefore be an indicator rather than a cause of disease.

The Mendelian randomization (MR) approach can overcome these limitations by using randomly allocated genetic variants as instrumental variables for studying the causal effects of modifying systemic iron status [2]. As germline genetic variation cannot be modified by the environment, this method is more robust to confounding and reverse causality. The aim of this study was to use MR to investigate the effect of genetically predicted systemic iron status on life expectancy.

## 2 Methods

### 2.1 Genetic instruments for iron status

As instrumental variables for systemic iron status, we selected single-nucleotide polymorphisms (SNPs) that had genome-wide significant associations (*P* < 5 × 10^−8^) with four biomarkers of iron status (serum iron, transferrin, transferrin saturation and ferritin) in a pattern with concordant effects on overall iron status [1,2,4,7]. The use of such a stringent significance threshold ensures that the SNPs may be modelled as strong instrumental variables for MR, and the association of the variants with all four biomarkers of iron status improves their validity as instruments for systemic iron status [1,2,4]. This approach identified three independent missense variants in genes implicated in iron homeostasis (rs1800562 – *HFE*, rs1799945 – *HFE*, rs855791 – *TMPRSS6*) [1,2,4,7]. Putative biological roles for these genes in iron homeostasis have been previously described [2]. Briefly, in the setting of excessive iron stores, *HFE* may inhibit iron absorption by inducing hepcidin production. In the setting of iron depletion, *TMPRSS6* may stimulate iron absorption by inhibiting hepcidin production.

### 2.2 Life expectancy outcomes

Large-scale genetic association studies on individual lifespan are not available. However, parental lifespan is a readily available phenotype that can be used as an outcome in a genetic association study because parents share half of their genetic code with their offspring [8]. As the primary outcome of the present analysis, we obtained genetic association estimates for the variants selected as iron status instruments with parental survival from a meta-analysis of the UK Biobank and LifeGen consortium (*n* = 1,012,240) [8]. These studies used a Cox proportional hazards model to estimate offspring SNP effects on parental survival. Effect sizes from this approach, using offspring genetic data, are half of the actual variant effect size in the parent [8], and were therefore doubled to reflect the expected genetic effects in parents. This approach effectively imputes the parental genotype data. The genetic effects from this study may be multiplied by ten to estimate the absolute change in lifespan years [8]. Data on the SNP-iron and SNP-longevity associations were harmonized by orienting effects to the same allele (Supplementary Table 1).

As a secondary outcome, we obtained genetic associations of these instruments with odds of individual (i.e. non-parental) survival to a sex and birth cohort-specific 90th percentile age vs. 60th percentile age (11,262 cases, 25,483 controls) [9]. As example survival percentiles, the 60th and 90th percentile ages in the 1920 US birth cohort correspond to 75 and 89 years for men and 83 and 102 years for women [9]. The genetic effects from this study are provided as log-odds of survival to the 90th vs. 60th percentile age.

All included genetic association studies were conducted in European populations and adjusted for principal components of ancestry, thereby minimizing confounding by population stratification. All studies received relevant ethical approval and participant consent and their data are publicly available, as detailed in the supporting citations [[7], [8], [9]].

### 2.3 Statistical analysis

For the three genetic variants, we used the ratio method to estimate the instrumental effect of higher iron status on life expectancy. This method divides the SNP-outcome effects by the SNP-exposure effects and uses first-order weights to obtain the standard error of the causal effect [10]. To pool the effects across all three variants, we implemented the random-effects inverse-variance weighted method [10]. This method regresses the SNP-outcome association on the SNP-exposure association and weights the effects by the inverse of the standard error of the SNP-outcome associations, with the intercept constrained at the origin [10]. We provide estimates that are scaled to a 1-standard deviation (SD) increase in iron status biomarker.

MR estimates may be biased by horizontal pleiotropy if the genetic variants proxying iron status influence longevity through a pathway independent of iron status. To test the null hypothesis of no pleiotropy, we calculated Cochran's Q for heterogeneity and used the *P-*value to assess the strength of evidence for pleiotropy [10]. We also performed analyses using the MR-Egger and weighted median methods, which can be robust to inclusion of pleiotropic variants [10]. As these analyses may be unreliable when only using three variants, we broadened the instrument selection criteria to include variants meeting the following criteria: i) associated with any 1 iron status biomarker at genome-wide significance and ii) associated with the other biomarkers in a pattern consistent with an effect on overall iron status, irrespective of the significance of association [4]. This approach identified three additional variants for use as genetic instruments for systemic iron status (Supplementary Table 2).

All analyses were implemented using the TwoSampleMR package of R [10].

## 3 Results

**Fig. 1:**
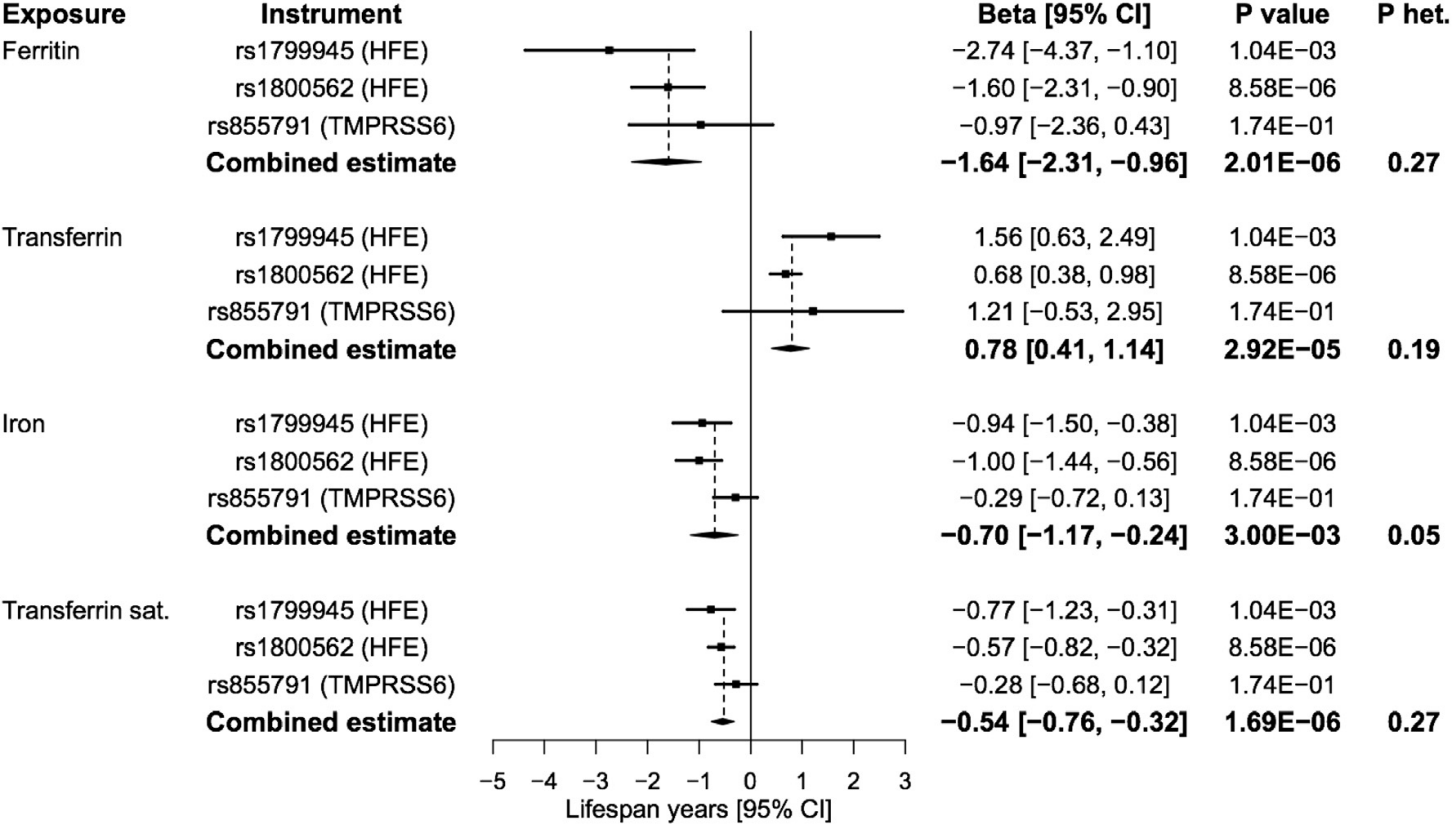
**Forest plot of Mendelian randomization estimates for the association between genetically predicted iron status biomarkers and lifespan (*n*** = **1,012,240).** Point estimates are expressed as change in lifespan years per standard deviation increase in genetically predicted iron status biomarker. ‘Combined estimate’ reports the effect estimated by the inverse-variance weighted method. CI: confidence interval; het: heterogeneity; sat: saturation.

**Fig. 2:**
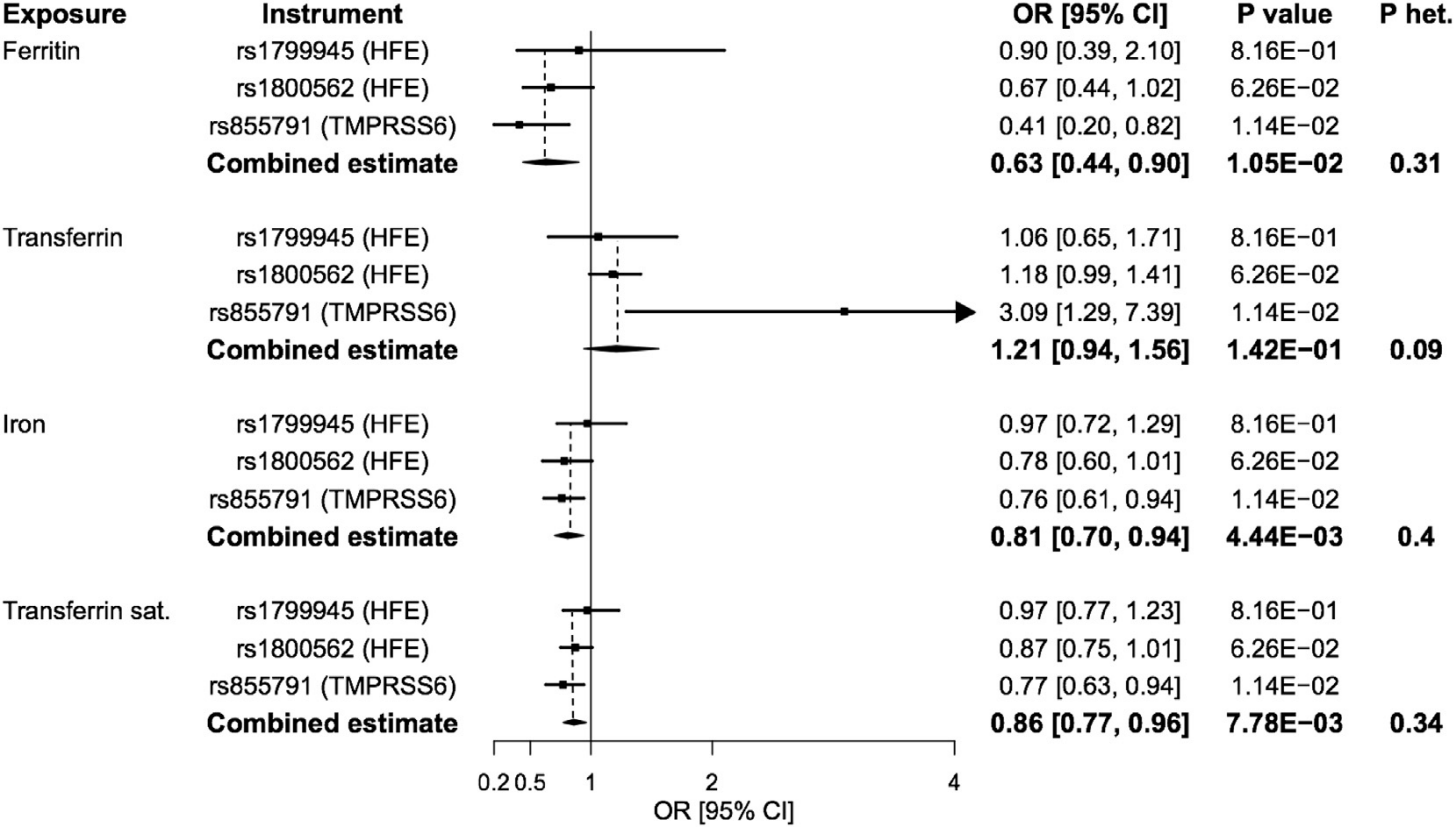
**Forest plot of Mendelian randomization estimates for the association between genetically predicted iron status biomarkers and survival to the 90th vs. 60th percentile age (11,262 cases/25,483 controls)**. Point estimates are expressed as the odds ratio (OR) for survival to the 90th vs. 60th percentile age per standard deviation increase in genetically predicted iron status biomarker. ‘Combined estimate’ reports the effect estimated by the inverse-variance weighted method. CI: confidence interval; het: heterogeneity; sat: saturation.

Genetically predicted higher iron status across all biomarkers was associated with reduced life expectancy (Fig. 1, Fig. 2). The association of a 1-SD increase in genetically predicted iron status biomarker with lifespan years was −0.70 for iron (95% confidence interval [CI] −1.17, −0.24; *P* = 3.00 × 10^−3^), −1.64 for ferritin (95% CI –2.31, −0.96; *P* = 2.01 × 10^−6^), −0.54 for transferrin saturation (95% CI −0.76, −0.32; *P* = 1.69 × 10^−6^), and 0.78 for transferrin (95% CI 0.41, 1.14; *P* = 2.92 × 10^−5^). The odds ratio for survival to the 90th vs. 60th percentile age was 0.81 for iron (95% CI 0.70, 0.94; *P* = 4.44 × 10^−3^), 0.63 for ferritin (95% CI 0.44, 0.90; *P* = 1.05 × 10^−2^), 0.86 for transferrin saturation (95% CI 0.77, 0.96; *P* = 7.78 × 10^−3^), and 1.21 for transferrin (95% CI 0.94, 1.56; *P* = 1.42 × 10^−1^). Individual variant MR estimates were all consistent with a deleterious effect of higher genetically predicted iron status on lifespan and longevity with no statistical evidence of heterogeneity (all *P* ≥ 0.05; Fig. 1, Fig. 2). Further sensitivity analyses using the MR-Egger and weighted median methods all provided consistent evidence with confidence intervals overlapping those obtained from the inverse-variance weighted method (Supplementary Tables 3 and 4). The MR-Egger intercept test did not provide evidence for unbalanced pleiotropy (all *P* ≥ 0.18).

## 4 Discussion

We found that higher genetically predicted iron status was associated with reduced life expectancy. This effect was consistent across all four biomarkers of systemic iron status, and across the outcomes of lifespan and longevity. The lack of heterogeneity across the genetic instruments and the consistency of evidence across statistical sensitivity analyses suggests that our findings are unlikely to be biased by pleiotropic effects of the variant used to proxy systemic iron status. While the association of genetically predicted transferrin levels with longevity in the secondary analysis was the only result where the confidence intervals overlapped the null, this is likely attributable to inadequate statistical power, and the direction of the point estimate was concordant with the other results.

Taken together, these findings suggest that associations between higher iron status and increased mortality risk reported in prior observational studies may reflect causal relationships [6]. Our findings suggest that, on average for the populations considered, any protective effects of an increase in iron status for certain outcomes are outweighed by deleterious effects on risk of other diseases. Prior MR studies have suggested that higher iron status reduces the risk of coronary artery disease [1] and hypercholesterolemia [2]. By contrast, MR studies have found deleterious effects of higher iron status on risk of skin infection [2], rheumatoid arthritis [3], and cardioembolic stroke [4]. Lifespan and longevity may be considered in relation to all these effects over the life course, thereby offering a holistic perspective on the consequence of intervening on a risk factor.

Our findings should be interpreted in context. Despite our efforts, the MR estimates may still be biased by pleiotropic effects of the genetic variants on life expectancy through pathways independent of iron status. Prior work has shown that the iron status raising allele at rs1800562 in *HFE* lowers low-density lipoprotein cholesterol levels, while the iron status raising allele at rs1799945 in *HFE* raises systolic and diastolic blood pressure [1]. However, we found consistent estimates in sensitivity analyses and little evidence of heterogeneity, suggesting that any bias attributable to these pleiotropic effects is unlikely to be large. Furthermore, this MR approach only considers the linear associations of small changes in genetically predicted iron status around the population mean, and cannot be extrapolated to infer the effect of changes in iron status outside of this normal range. As genetic variation causes lifelong changes in iron status, these results cannot be extrapolated to predict the effect of a discrete clinical intervention that modifies iron status. This analysis was conducted using data from European-ancestry population-based studies, and may not generalize to other populations. Finally, this study design does not inform on the biological mechanisms by which systemic iron status influences life expectancy.

In conclusion, our genetic evidence suggests that an increase in systemic iron status around the population mean may reduce life expectancy. While randomized-controlled trials are required to provide definitive evidence of clinical effect, and further research is required to validate the clinical implications of our findings, caution may be prudent when supplementing iron without a clear clinical indication, such as in individuals with normal iron status.

## Role of the funding sources

The funding source had no role in the design, acquisition of data, analysis, interpretation or write up of this study.

## Funding

DG is funded by the Wellcome Trust 4i Programme (203928/Z/16/Z) and British Heart Foundation Centre of Research Excellence (RE/18/4/34215) at Imperial College London.

## Author contributions

ID and DG designed the study, performed statistical analyses, interpreted results, wrote the manuscript, edited the manuscript for intellectual content, and take responsibility for the integrity of the study.

## Conflict of interest

DG is employed part-time by Novo Nordisk. ID has no conflicts of interest to declare.

## References

[1] Gill D., Del Greco M.F., Walker A.P., Srai S.K.S., Laffan M.A., Minelli C. (2017). The effect of iron status on risk of coronary artery disease. Arterioscler Thromb Vasc Biol.

[2] Gill D., Benyamin B., Moore L.S.P., Monori G., Zhou A., Koskeridis F. (2019). Associations of genetically determined iron status across the phenome: a mendelian randomization study. PLoS Med.

[3] Yuan S., Larsson S. (2020 Oct). Causal associations of iron status with gout and rheumatoid arthritis, but not with inflammatory bowel disease. Clin Nutr.

[4] Gill D., Monori G., Tzoulaki I., Dehghan A. (2018). Iron status and risk of stroke: a Mendelian randomization study. Stroke.

[5] Bailey R.L., Gahche J.J., Lentino C.V., Dwyer J.T., Engel J.S., Thomas P.R. (2011). Dietary supplement use in the United States, 2003–2006. J Nutr.

[6] Ellervik C., Tybjærg-Hansen A., Nordestgaard B.G. (2011). Total mortality by transferrin saturation levels: two general population studies and a metaanalysis. Clin Chem.

[7] Benyamin B., Esko T., Ried J.S., Radhakrishnan A., Vermeulen S.H., Traglia M. (2014). Novel loci affecting iron homeostasis and their effects in individuals at risk for hemochromatosis. Nat Commun.

[8] Timmers P.R., Mounier N., Lall K., Fischer K., Ning Z., Feng X. (2019). Genomics of 1 million parent lifespans implicates novel pathways and common diseases and distinguishes survival chances. Elife.

[9] Deelen J., Evans D.S., Arking D.E., Tesi N., Nygaard M., Liu X. (2019). A meta-analysis of genome-wide association studies identifies multiple longevity genes. Nat Commun.

[10] Hemani G., Zheng J., Elsworth B., Wade K.H., Haberland V., Baird D. (2018). The MR-Base platform supports systematic causal inference across the human phenome. Elife.

